# The Biocompatibility of Degradable Magnesium Interference Screws: An Experimental Study with Sheep

**DOI:** 10.1155/2015/943603

**Published:** 2015-01-31

**Authors:** Ulrich Thormann, Volker Alt, Lydia Heimann, Cyrille Gasquere, Christian Heiss, Gabor Szalay, Jörg Franke, Reinhard Schnettler, Katrin Susanne Lips

**Affiliations:** ^1^Laboratory for Experimental Trauma Surgery, Justus-Liebig University Giessen, Kerkrader Straße 9, 35394 Giessen, Germany; ^2^Department of Trauma Surgery Giessen, University Hospital of Giessen-Marburg, Justus-Liebig University Giessen, Rudolf-Buchheim-Straße 7, 35392 Giessen, Germany; ^3^aap Biomaterials GmbH, Lagerstraße 11-15, 64807 Dieburg, Germany; ^4^Department of Trauma and Orthopedic Surgery, Elbe Kliniken Stade, Bremervörder Straße 111, 21682 Stade, Germany

## Abstract

Screws for ligament reconstruction are nowadays mostly made of poly-L-lactide (PLLA). However, magnesium-based biomaterials are gathering increased interest in this research field because of their good mechanical property and osteoanabolic influence on bone metabolism. The aim of this pilot study was to evaluate the biocompatibility of an interference screw for ligament reconstruction made of magnesium alloy W4 by diecasting and milling and using different PEO-coatings with calcium phosphates. PLLA and titanium screws were used as control samples. The screws were implanted in the femur condyle of the hind leg of a merino sheep. The observation period was six and twelve weeks and one year. Histomorphometric, immunohistochemical, immunofluorescence, and molecular biological evaluation were conducted. Further TEM analysis was done. In all magnesium screws a clinically relevant gas formation in the vicinity of the biomaterial was observed. Except for the PLLA and titanium control samples, no screw was fully integrated in the surrounding bone tissue. Regarding the fabrication process, milling seems to produce less gas liberation and has a better influence on bone metabolism than diecasting. Coating by PEO with calcium phosphates could not reduce the initial gas liberation but rather reduced the bone metabolism in the vicinity of the biomaterial.

## 1. Introduction

In orthopedic and trauma surgery, absorbable implants are of major interest because it would make implant removal after bone healing redundant. Furthermore, sufficient fixation techniques are necessary for ligamentous (e.g., ACL) reconstruction, which also allow a complete osseous integration of the tendon by a slow degradation process. Currently, interference screws made of degradable polymers like PLLA are commonly used for ligamentous fixation by blocking the implanted tendon in the drill hole of the cancellous bone [1]. In order to improve the mechanical strength of screws, metal implants are in the focus of current research. Magnesium and magnesium alloys are of special interest as structural materials since their high specific strength enables them to be applied as implants. In particular the related elastic modulus of magnesium (41–45 GPa), compared to natural bone (3–20 GPa), makes this metal an ideal bone substitute or osteosynthesis material by decreasing the “stress-shielding” effect [2]. In addition, magnesium and magnesium-alloys support the development of stromal cells towards osteoblasts and subsequently stimulate the production of extracellular matrix [3].

A major problem is that magnesium is low corrosion resistant and may undergo an environmentally induced degradation [4]. In the presence of body fluid, forming an electrolytic and aqueous environment, pure magnesium corrodes fast and sequentially releases hydrogen gas [5, 6]. Thereby, the mechanical integrity of the implant decreases and the high amount of gas stresses the surrounding host tissue [2]. To control this degradation process several approaches have been undertaken. On one hand alloying improves the mechanical properties and can reduce the degradation process; on the other hand surface treatments and coatings have been of major interest to address the ability of a controlled degradation [2, 6].

Several studies have investigated different magnesium-alloys, like AZ91, LAE442, RS66, WE43, or W4 on their degradation behavior [7–10]. Also* in vivo* studies with different implants made of magnesium-alloys have been performed and a clinical relevant gas formation with loosening of the implant material has never been discussed. Castellani et al. [11] have published a study in rats where rods based on WE43 were implanted in cortical bone and had a better osseointegration and consequently a higher mechanical stability in host bone than titanium pins after 4, 12, and 24 weeks. Recently, Waizy et al. [12] showed a moderate gas formation without impairment in osseous integration in MgYREZ-screws, which is comparable to WE43 (93 wt.% magnesium, 4 wt.% yttrium, and 3 wt.% rare-earth). Bobe et al. [7] also attested a good biocompatibility without clinical relevant gas formation of a W4 (96 wt.% magnesium, 4 wt.% yttrium) scaffold. The W4 alloy, containing 96 wt.% magnesium and 4 wt.% yttrium, seems to have a good cytocompatibility beside a high corrosion-resistance and shows additionally improved mechanical properties compared to the W43 alloy [13].

In order to further reduce the degradation process, different coating procedures, such as microarch oxidation (MAO) with hydroxyapatite [14] or other calcium phosphates [6, 15–17], were on trial and showed a decreased absorption of the material. In all of these studies a gas formation surrounding the implant material was described but considered as being not clinically relevant. Regarding the fact that fixation of an implanted ligament requires a good primary stability, especially over the first 6–12 weeks in ACL-reconstruction, even a slight loosening of the fixation due to a minor surrounding gas formation is crucial.

If the degradation process and the consecutive gas liberation of magnesium alloys were to be under control, magnesium interference screws would be advantageous in comparison to PLLA and more so to titanium screws, because of their better mechanical properties and osteoinductive potential.

To the authors best knowledge there is no existing study, which addresses the degradation process of an absorbable, coated W4-magnesium interference screw during the first weeks after implantation with special emphasis on the implant interface region.

Aim of this pilot study was to investigate the biocompatibility of W4 interference-screws with different coatings of calcium phosphates and two different production methods that were implanted into the cancellous bone of the femur condyle of a sheep.

## 2. Materials and Methods

### 2.1. Animal Model

After approval of the animal application by the local authorities according to the Protection of Animals Act (reference number: V 54–19 c 20/15–D 20/Anz. 04), we operated on 15 female merino sheep at both sides of the hind leg in general anesthesia. Each animal was randomly assigned to the implanted biomaterial and to three different time periods, 6 weeks (*n* = 6), 12 weeks (*n* = 6), and one year (*n* = 3). Under aseptic conditions and after draping the knee region in a sterile manner, we exposed the medial aspect of the femur condyle without opening the knee joint by dissecting the septum intermusculare. Using an 8 mm drill bit (Fa. DePuySynthes, Tuttlingen, Germany) we drilled by water-cooling an 8 mm wide hole in the femurs' distal epiphysis. Afterwards we screwed in the interference screw with a size of 8 × 23 mm made of different materials (Table 1).

Finally the wound closure was performed in layers by absorbable stiches. A spray dressing was applied to prevent contamination of the wound. The animals were kept individually for one week to ensure wound healing and for the rest of the observation period in a flock on a nearby meadow with hay and water ad libitum. After 6 and 12 weeks and 12 months euthanasia was performed and the specimen underwent further examination.

### 2.2. Bone Substitution Materials

The interference screws were made of (a) PLLA (poly-L-lactide acid; Fa. Arthrex) or (b) titanium (Fa. aap Biomaterials GmbH, Dieburg) representing two control groups. The screw to be tested was made of a magnesium alloy, containing 96 wt.-% Mg, 4 wt.-% Yttrium, <0.25 wt.-% rare earth (W4) (Fa. aap Biomaterials GmbH, Dieburg). The manufacture was done either by diecasting or by milling the screw out of an extruded cast part. To control degradation we generated a layer of calcium phosphates (Table 1). Coating was performed by plasma electrolytic oxidation (PEO). PEO also often denoted as microarc oxidation (MAO) is a chemical conversion of the substrate's surface in an oxide or ceramic layer that occurs normally in an aqueous electrolyte under plasma discharge and sparkling conditions [18–22]. The coatings thus obtained have shown excellent mechanical properties. Therefore, PEO has long been used for wear protection [23–25], corrosion protection [26, 27], and thermal protection [28–30] of light metals and their alloys. Recent studies investigated the suitability of PEO coating on magnesium alloys [6, 14, 16, 31]. The coating was conducted with different bisphosphonates and calcium phosphates (CaO), CaHPO_4_, Mg(OH)_2_, nanohydroxyapatite, Ca(H_2_PO_4_), and Na-silicate (Na_2_O·3SiO_2_). The thickness of the layer was determined by the exposition time.

### 2.3. Macroscopic Evaluation and Histology

Directly after removing the femur condyle, the specimen was cut into slices of 3 mm thickness and documented with a digital camera. Subsequently the pictures were used for macroscopic evaluation.

Slices (3 mm) for histology were fixed in 4% phosphate buffered paraformaldehyde (Carl Roth, Karlsruhe, German) and either used for preparation of demineralized paraffin sections or undemineralized grindings. Samples for paraffin embedding were decalcified in 10% ethylene diamine tetra acetic acid (Merck, Darmstadt, Germany) in 0.281 M Tris-buffer, dehydrated with an increasing series of ethanol and xylene, embedded in paraffin, and cut with a thickness of 4-5 *μ*m at a rotation microtom (RM 2155, Leica, Bensheim, Germany) and stained with toluidine blue (TB) and hematoxylin and eosin (HE, Merck). Undemineralized samples were embedded in Technovit 9100 NEU according to the manufacturer's protocol (Heraeus Kulzer, Hanau, Germany). Technovit grindings with a thickness of approximately 20 *μ*m were performed with a cutting-grinding system (Exact, Nordersted, Germany), stained with TB and HE, cover-slipped, and evaluated with a light microscope (Axiophot-2, Zeiss, Jena, Germany) equipped with a digital camera (DC 500, Leica, Bensheim, Germany).

### 2.4. Enzyme and Immunohistochemistry and Double-Labelling Immunofluorescence

Enzyme histochemistry was performed to detect Tartrate-resistant acidic phosphatase (TRAP) as well as chloracetate esterase (CAE) to determine osteoclasts, macrophages, and infiltrating neutrophils, respectively. For TRAP histochemistry deparaffinized sections were washed in 0.1 M acetate buffer (pH 5.2), incubated in a solution of Naphthol AS-TR phosphate (Sigma-Aldrich, Steinheim, Germany), di-sodium-tartrate-dihydrate (Merck), and fast red TR salt (Sigma-Aldrich) in acetate buffer for 30 min at 37°C. After being washed in distilled water nuclei were counterstained with hematoxylin and cover-slipped with Kaiser's glycerol gelatin (Merck).

For chloracetate esterase (CAE) histochemistry deparaffinized sections were incubated for 20 minutes (min) in a solution containing naphthol AS-D chloracetate (Sigma-Aldrich), N-N dimethylformamide, 4% sodium nitrite, and 4% new fuchsin in 2 N hydrochloric acid. As a result neutrophils showed a red colour. Sections were counterstained with haematoxylin before cover-slipped with Kaiser's glycerol gelatin.

For conduction of immunohistochemistry deparaffinized sections were washed in a solution of 0.025% Trition-X-100 (Merck) in Tris-NaCl buffer (TBS, pH 7.4). To block the endogenous peroxidase sections were incubated with 3% H_2_O_2_ in TBS for 5 min. After washing in TBS, sections were incubated with the primary antibodies in dilution buffer (Dako, Hamburg, Germany). The following were used: (a) rabbit anti-human collagen 1 antibody (Biomex, Heidelberg, Germany, diluted 1 : 500), (b) mouse anti-human CD 68 antibody (Dako, diluted 1 : 50), (c) mouse anti-human osteocalcin antibody (R&D Systems, Wiesbaden, Germany), and (d) mouse anti-human smooth muscle actin (clone 1A4, Dako, diluted 1 : 400). Afterwards, sections were washed in TBS, incubated for 30 min with biotinylated goat anti-rabbit secondary antibody (Vector Laboratories Inc., Burlingame, CA, USA, diluted 1 : 500) or biotinylated horse anti-mouse secondary antibody (Dako, diluted 1 : 150), and subsequently treated for 30 min with Streptavidin-Biotin-Peroxidase Complex (Vector Laboratories Inc.). Staining was rendered visible by incubation with peroxidase Nova-Red substrate kit (Vector Laboratories Inc.) according to the manufacturer's instructions of an incubation time of 5 min. Sections were counterstained with hematoxylin, cover-slipped with DePex (Serva, Heidelberg, Germany), and evaluated with the light microscope. As negative control immunohistochemistry was performed without incubation of first antibody.

For immunofluorescence double labelling deparaffinized sections were incubated with mouse anti-human smooth muscle actin (clone 1A4, Dako, diluted 1 : 400) for 1 h at room temperature. After washing, sections were covered with biotinylated horse anti-mouse secondary antibody (Dako, diluted 1 : 400) and subsequently incubated with DyLight-488-Streptavidin (KPL, Baltimore, Maryland, USA) and DAPI (Höchst 33258, Sigma, Taufkirchen, Germany). Sections were cover-slipped with ProLong Gold antifade reagent (Life Technologies, Regensburg, Germany) and evaluated with an inverted fluorescence microscope (Olympus IX81, Olympus Deutschland GmbH, Hamburg, Germany).

### 2.5. Real-Time RT-PCR

One slice of femur condyle was placed in RNAlater (Qiagen, Hilden, Germany), and 100 mg of tissue at the implant interface was cut out and used for extraction of total RNA with the RNeasy Lipid Tissue Mini Kit (Qiagen) according to the manufacturer's protocol. After removal of contaminating genomic DNA the RNA was reverse-transcribed with the QuantiTect Reverse Transcription Kit (Qiagen) as described by the manufacturer. Afterwards real-time RT-PCR was performed in the LightCycler (Roche, Grenzach, Germany) using the QuantiFast SYBR Green PCR Kit (Qiagen). Therefore 1 *μ*L of cDNA was added to 5 *μ*L Mastermix, 0.2 *μ*L forward and reverse primer (Eurofins MWG, Ebersberg, Germany, Table 2), and 3.8 *μ*L RNase free water. The samples were incubated for 5 min at 95°C, followed by 40 cycles of 10 seconds (sec) heating at 95°C and annealing for 30 sec at 60°C. Subsequently the PCR product was checked by melting curve, gel electrophoresis, and sequencing. As a negative control, reverse transcription was conducted without enzymes and template was replaced by water. The LightCycler Software measured the crossing points (Cp) for each graph. ΔCp values were calculated by subtraction of the sample Cp values and Cp of the reference gene *β*2-microglobulin (*β*MG).

### 2.6. Statistical Analysis

The SPSS-software (version 21, SPSS Institute Inc., Chicago, IL, USA) was used for statistical analysis. Comparison of nonparametric independent samples was conducted with the Kruskal-Wallis, and subsequently the Mann-Whitney test was used to investigate the differences between the groups. The value of *P* ≤ 0.05 was considered significant.

### 2.7. Transmission Electron Microscopy (TEM)

Samples for TEM were fixed for 6 h in a solution containing 2% paraformaldehyde, 2% glutardialdehyde, and 0.02% picric acid in 0.1 M phosphate buffer (pH of 7.2). After being washed in 0.1 M cacodylate buffer samples were incubated for 2 h in 1% osmium tetroxide solution, dehydrated with an increasing series of ethanol and xylene, and were allowed to polymerize in Epon (Serva). Samples were cut with a thickness of 500 nm, stained with toluidine blue and Safranin-O, and evaluated with a light microscope. Furthermore ultrathin sections with a thickness of 60–80 nm were cut, contrasted with uranyl acetate and lead citrate, and analyzed with a TEM (LEO EM 912, Zeiss, Oberkochen, Germany) equipped with a slow scan CCD camera.

## 3. Results

### 3.1. General Considerations

All animals survived the surgical procedure and the postoperative observation time. No adverse events were noticed and the euthanasia was conducted as planned. Neither could significant gas formation be detected under the skin nor were signs of infection present.

### 3.2. Macroscopic Evaluation and Histology

**Figure 1:**
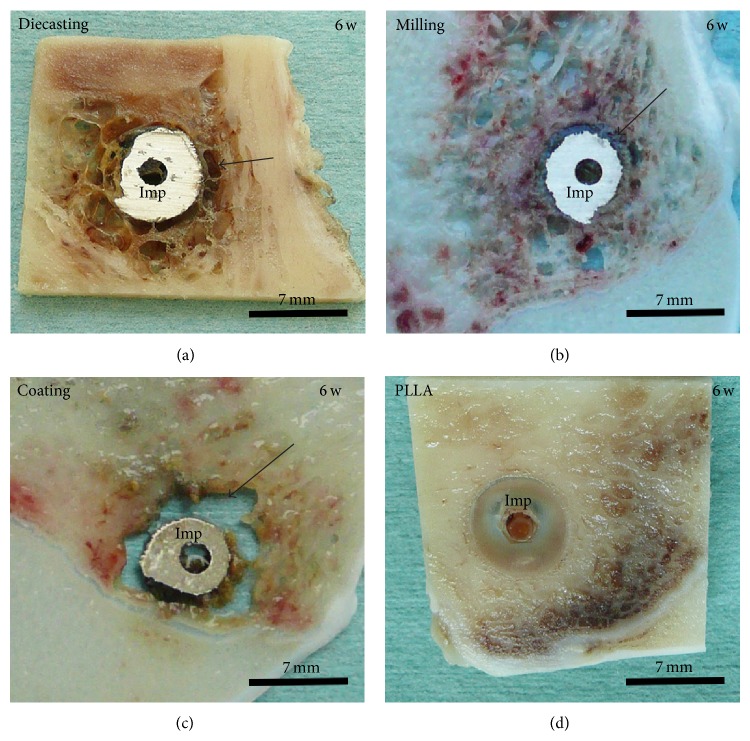
Macroscopic evaluation, 6 weeks (w) after operation. Magnesium implants generated (a) by diecasting and (b) by milling and (c) modified with a coating were surrounded by gas voids (arrow). (d) PLLA screws were used as control samples. Imp: implant.

All slices with Mg-implants—diecast, milled and coated—presented gas voids surrounding the whole implant and spreading into the ambient cancellous bone tissue after six weeks (Figure 1). A strong connection to the bone tissue was missing and therefore an implant loosening was provoked. The control implants consisting of titanium and PLLA had a good osseous integration (Figure 1(d)).

**Figure 2:**
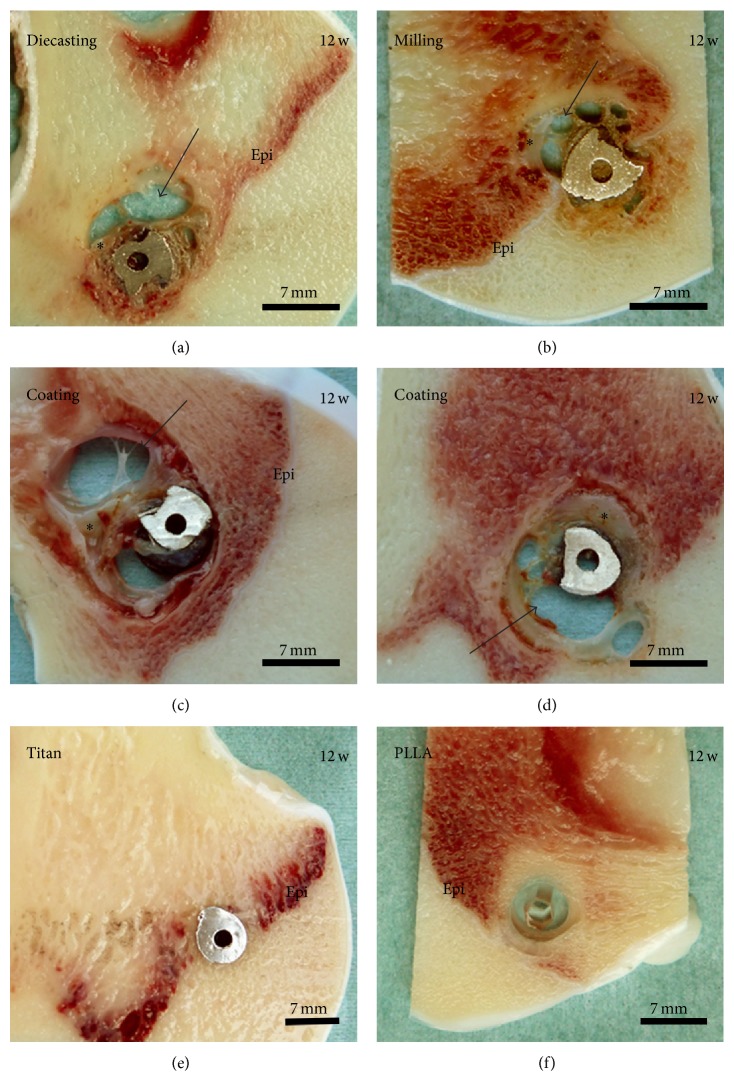
Macroscopic evaluation, 12 weeks (w) after operation. Magnesium implants generated (a) by diecasting and (b) by milling and (c)-(d) modified with a coating of a thin layer of calcium phosphate were surrounded by gas voids (arrow) and soft tissue (star). (c) 150 sec coating, (d) 300 sec coating. (e) Titanium and (f) PLLA screws were used as control samples. Epi: epiphyseal plate.

At the 12-week follow-up time the control implants were well osseously integrated whereas all Mg-implants were still surrounded by gas voids (Figure 2). In addition some soft tissue was found at the interface. When comparing the different Mg implants it seemed that the implant with thicker calcium phosphate coating (exposition time of 300 sec, Figure 2(d)) presented more soft tissue and less gas voids at its interface compared to the implant with thinner coating (150 sec, Figure 2(c)). In general, the coated Mg implants formed more gas voids than the implants generated by diecasting (Figure 2(a)) and milling (Figure 2(b)).

**Figure 3:**
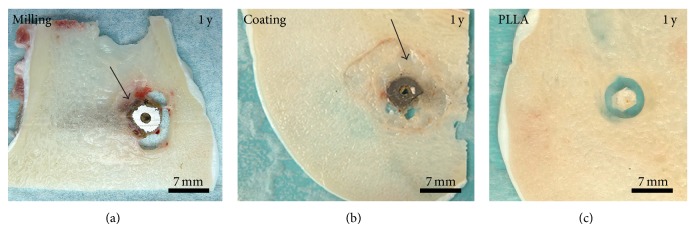
Macroscopic evaluation, 1 year (y) after operation. Magnesium implants generated (a) by milling and (b) modified with a coating were surrounded by corrosion product (star), gas voids, and soft tissue (arrow). (c) PLLA screws were used as control samples.

Furthermore, voids filled with gas were also observed after one-year follow-up time (Figure 3). At this point the diecast samples with coating showed less gas voids than the samples with Mg implant produced by milling. However, the control samples still demonstrated the best osseous integration (Figure 3(c)).

**Figure 4:**
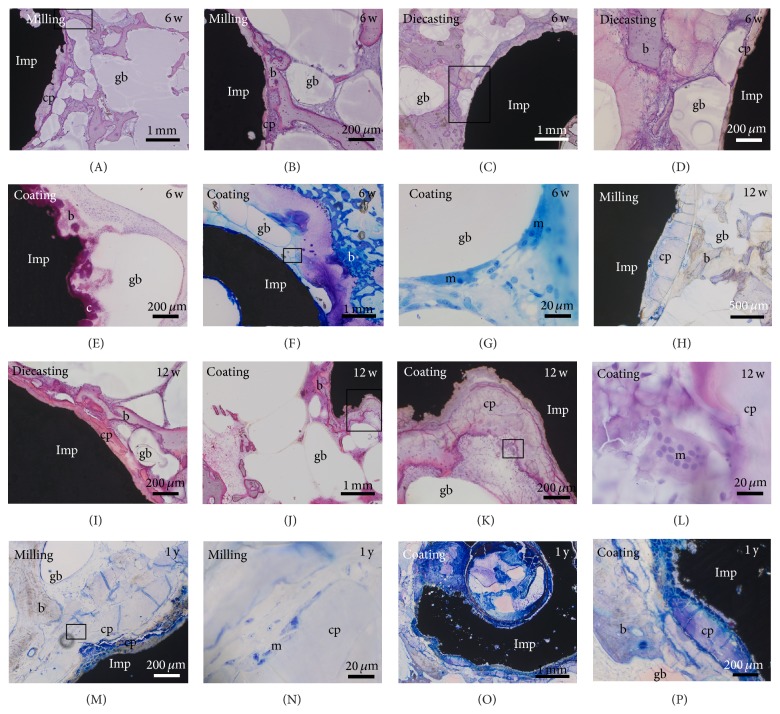
Degradation of Mg implants. The amount of corrosion product (cp) was not significantly different between the (A)–(G) 6- and (H)–(L) 12-week (w) and (M)–(P) 1-year (y) follow-up times. Implants (Imp) were surrounded by bone (b), granulation tissue, gas voids (gb), and macrophages (m). ((L), (N)) Multinucleated macrophages were also located at the Mg corrosion product. (I) Hardly any residue was found from the calcium phosphate coating (c). Square: detail shown in higher magnification in the subsequent picture.

Degradation was identified by surrounding Mg corrosion product, the rough surface of the implant, and changes in shape and decreasing size. Degradation of Mg had already started after 6 weeks, increasing slightly until the one-year check but had not been finished at this point (Figures 1–4). Mg corrosion product seemed unavoidable by cells. Sometimes multinucleated macrophages (Figures 4(L) and 4(N)) or fibroblast-like cells were found at the surface of Mg corrosion product. No significant differences were observed in the amount of corrosion product or cellular growth on the surface of corrosion product between the different Mg implants (Figure 4).

**Figure 5:**
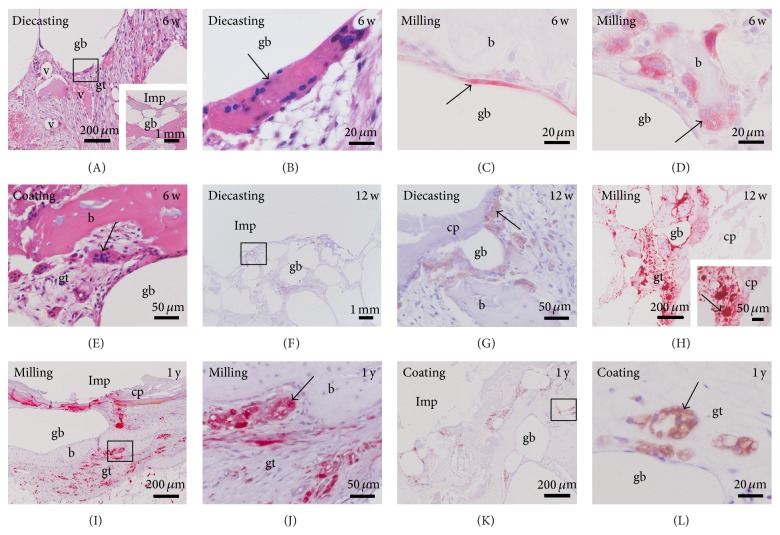
Location of multiple macrophages. High amounts of multiple macrophages (arrow) were found in the surrounding of Mg implants (A)–(E) 6 and (F)–(H) 12 weeks (w) and (I)–(L) 1 year (y) after implantation. Multiple macrophages were identified by (A, B, and E) their size and multiple nuclei (HE staining) as well as by (C)-(D), (I)-(J) TRAP and (F)-(G), (K)-(L) CD68 labeling. They were situated at the surface of gas voids (gb), bone (b), and Mg corrosion product (cp) as well as in the granulation tissue (gt) surrounding the gas voids. Imp: empty space that was created by removing the implant before preparation of paraffin sections. Square: detail shown in higher magnification in subsequent picture, and v: vessels. Inset in (A) overview of A. Inset in (H) higher magnification of TRAP positive cells at the corrosion product.

The gas voids were surrounded by connective tissue that included new-formed bone tissue, outgrowing vessels, and a high amount of multinucleated macrophages (Figures 5(A) and 5(B)).

### 3.3. Enzyme and Immunohistochemistry and Double-Labelling Immunofluorescence

Multinucleated macrophages and osteoclasts were determined with TRAP enzyme histochemistry (Figures 5(C), 5(D), and 5(H)–5(J)) as well as with CD68 immunohistochemistry (Figures 5(F), 5(G), 5(K), and 5(L)). A high number of TRAP and CD68 positive cells were found surrounding the gas voids (Figures 5(A)–5(C)). Some multinucleated macrophages were located at the Mg corrosion product that was found at the implant interface (Figures 5(F), 5(G), and 5(I)). Only a few osteoclasts were situated at the trabecular surface (Figure 5(D)). Most multinucleated macrophages were found lost in the granulation tissue surrounding the gas voids (Figures 5(E), 5(H), 5(J), and 5(L)). No differences were detected in the amount of TRAP and CD68 positive cells between the different Mg implants and at the different postoperative follow-up times (Figure 5). Control samples with an implant of titanium or PLLA exhibited a much smaller number of multinucleated macrophages and osteoclasts (data not shown).

**Figure 6:**
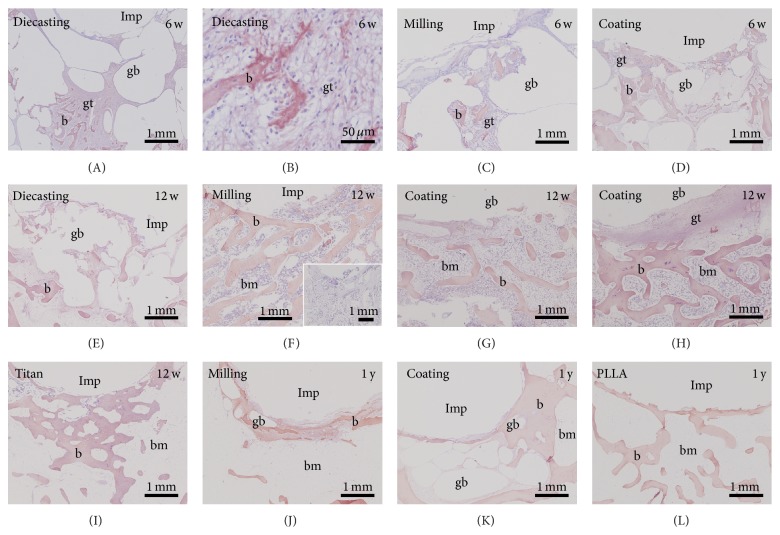
Collagen-1 immunohistochemistry did not show any difference in the amount and areal organization comparing the different implants and follow-up times. w: weeks, gb: gas void, b: bone, bm: bone marrow, gt: granulation tissue, and Imp: empty space that is created by removing the implant before preparation of paraffin sections. Inset in (F): negative control omitting incubation of primary antibody.

New-formed trabeculae were identified by collagen 1 immunohistochemistry. No obvious alterations between the different Mg implants and control samples (titanium and PLLA) were observed at the investigated postoperative times (Figure 6).

Osteoblasts were identified by osteocalcin immunohistochemistry that determined some reactivated osteoblasts no matter what implants were used (data not shown).

**Figure 7:**
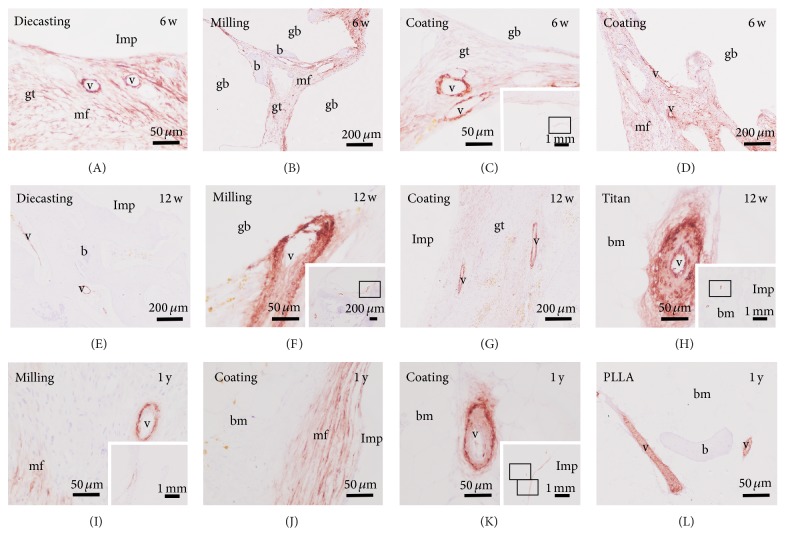
Location of blood vessels by anti-smooth muscle actin (ASMA) immunohistochemistry. Vessels (v) and myofibroblast (mf) are identified by their red staining in the granulation tissue (gt) located between the gas voids (gb) and at the implant (Imp) interface at the 6-week (w) follow-up time (A–D). After 12 weeks the amount of ASMA staining was reduced (E–H). In the PLLA (L) and titanium (H) controls no myofibroblast was labeled and vessels were mainly found in erosions lacunae and bone marrow (bm). The amount of myofibroblasts was increasingly reduced in the samples with Mg implants. Only a limited number of blood vessels were found mainly in bone marrow. Insets in (C), (F), (H), (I), and (K) overviews.

Vessels were determined with immunolabelling using a primary antibody against smooth muscle actin (ASMA), which also labelled myofibroblasts (Figure 7). A similar amount and distribution of vessels were observed in samples of the 6-week follow-up time with the different Mg implants (Figures 7(A)–7(D)). The vessels were located in the granulation tissue at the interface as well as around the gas voids. At the same location a high amount of ASMA-immunopositive myofibroblasts were detected. In the control samples with insertion of PLLA and titanium implants no myofibroblasts were found and the vessels were mainly situated in the erosions lacunae and bone marrow space. After the 12-week follow-up period vessels and myofibroblast were detected at the same location but in a reduced number (Figures 7(E)–7(H)). In samples with Mg implants generated by diecasting myofibroblasts were not present any more. After the 1-year follow-up a very limited number of myofibroblasts were found at the interface of Mg milling implants whereas several immunopositive cells were found near the Mg coating implants. Blood vessels were only located in the bone marrow and their number was declined to a very low figure (Figures 7(I)–7(K)). In the control samples with PLLA implant no myofibroblast was found and vessels were only determined in erosion lacunae and bone marrow space (Figure 7(L)).

**Figure 8:**
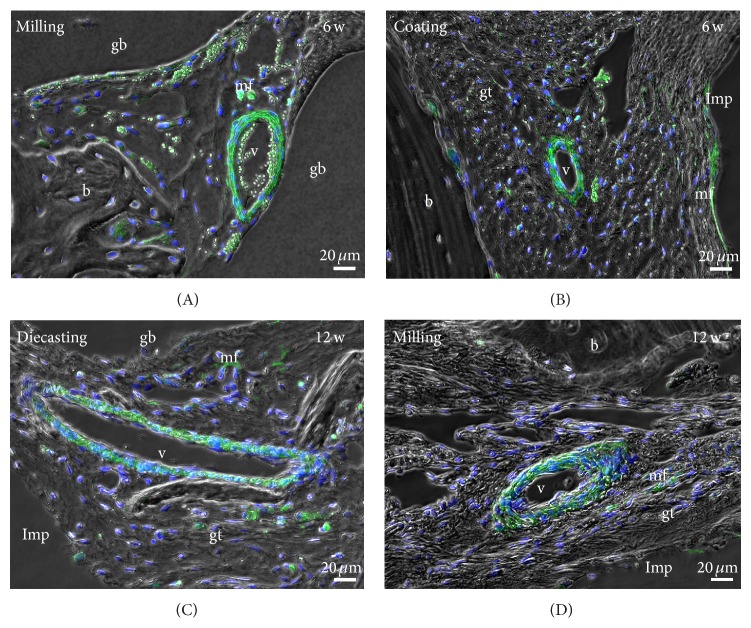
Double-labelling immunofluorescence with anti-smooth muscle actin (ASMA, green) and nuclear staining DAPI (blue) verified the results of ASMA immunohistochemistry. b: bone, gb: gas void, gt: granulation tissue, v: blood vessel, mf: myofibroblast, and Imp: empty space that is created by removing the implant before preparation of paraffin sections.

Results of ASMA were verified by immunofluorescence in colocalization with DAPI as a marker for nuclei (Figure 8).

**Figure 9:**
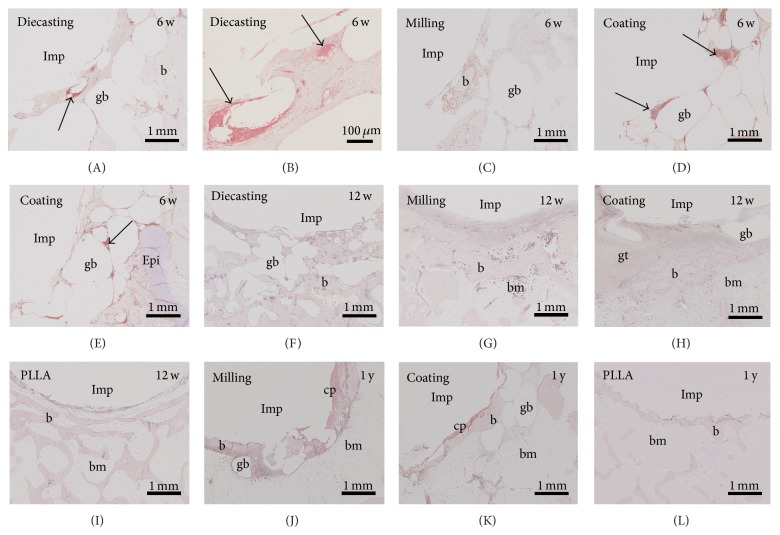
Chloracetatesterase enzyme histochemistry demonstrated (A)–(E) accumulation of erythrocytes (arrow) at implant interface of the different Mg screws ((A)-(B) diecasting, (C) milling, (D)-(E) coating) without any alterations between them. (F)–(I) At the postoperative follow-up times of 12 weeks (w) and (J)–(L) 1 year (y) no accumulation of erythrocytes was found. Infiltration of neutrophil granulocytes was not detected. Imp: empty space that is created by removing the implant before preparation of paraffin sections, gb: gas void, b: bone, epi: epiphyseal plate, bm: bone marrow, gt: granulation tissue, and cp: corrosion product.

Infiltrating neutrophil granulocytes as a sign of a foreign body reaction or an infection were identified using the CAE histochemistry. No accumulation of neutrophil granulocytes was found in the test groups or in the control samples at any time point (Figure 9).

### 3.4. Real-Time RT-PCR

**Figure 10:**
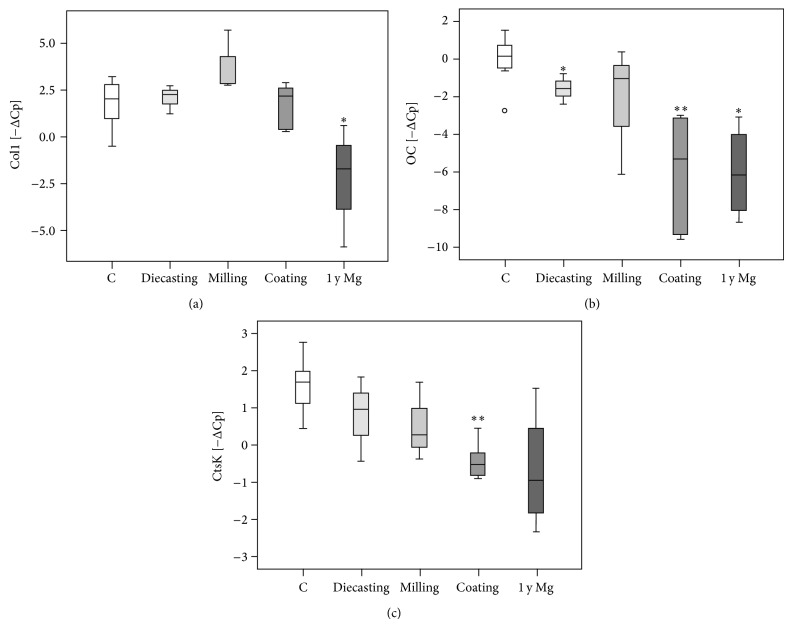
Real-time RT-PCR. (a) Significant reduction of collagen-1 expression (Col1) 1 year (y) after insertion of Mg implants. (b) Osteocalcin (OC) expression was reduced after insertion of Mg implants generated by diecasting, additional coating and at 1-year follow-up time. (c) Cathepsin K mRNA expression was reduced for Mg implants with a coating and after 1 year after operation. Minus delta-Cp values are presented as box plot with the median indicated by a solid line within the box. Data beyond 1.5 x of the interquartile range of the median are marked with a small circle. ^*^ *P* ≤ 0.05, ^**^ *P* ≤ 0.01.

All investigated bone substitution materials were surrounded with tissue that expressed collagen I*α*1 (col1), osteocalcin (OC), and cathepsin K (CtsK) mRNA. Using real-time RT-PCR we added the ΔCp values of PLLA and titan samples of the postoperative time points 6 and 12 weeks (w) as controls (*n* = 5) and compared them with the Mg screws produced by diecasting (*n* = 3), milling (*n* = 3), and coating (*n* = 6). For the animals with a postoperative duration of 1 year the samples with Mg implants (*n* = 4) were added and compared to the control samples (*n* = 2). The col1 values showed no significant differences when applying the statistical Mann-Whitney test. OC mRNA was significantly decreased in the tissue surrounding the diecasting implants as well as the coated implants by comparison to the control samples. The CtsK mRNA expression was significantly reduced in the group with the coated implants compared to the control group. Comparing the control samples of the different postoperative time points we could not determine any significant differences; therefore they were summed up to the control-total group (c-total) in a second set of statistical analysis. When the c-total group was compared with the diecasting, the milling, the coating, and the 1-year Mg-group, significant differences were detected for all 1-year Mg samples, for OC mRNA of the diecasting group and for OC and CtsK mRNA of the coating groups (Table 3, Figure 10).

### 3.5. Transmission Electron Microscopy (TEM)

**Figure 11:**
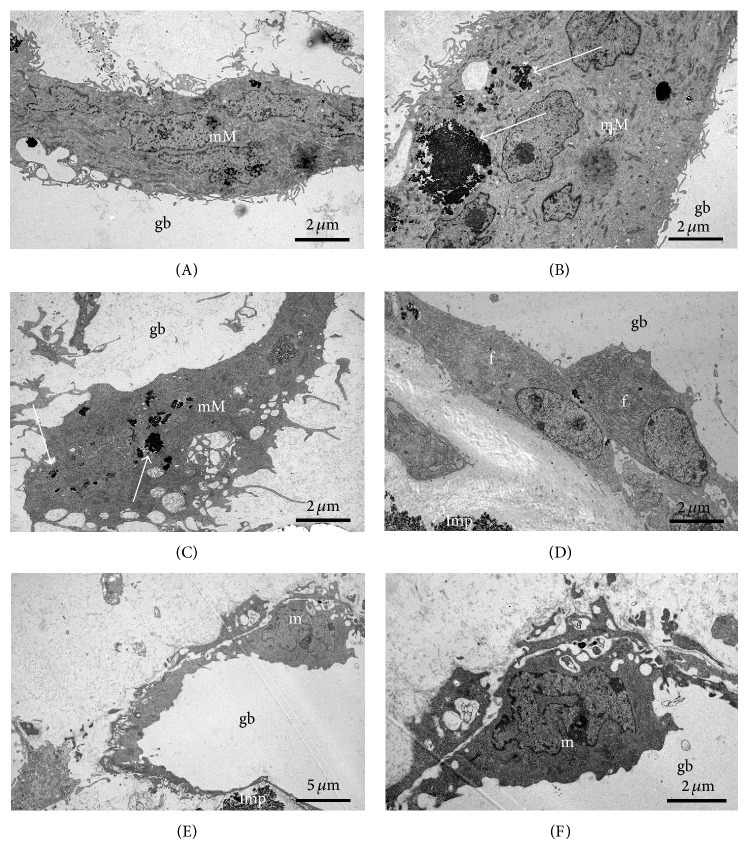
Transmission electron microcopy, 6 weeks after operation. (A–C) Multinucleated macrophages (mM) are located at the surface of gas voids (gb), contain resorbed implant material (arrow), and hold processes (p). (D–F) Gas voids are also covered by fibroblast-like cells (f) and uninucleated macrophages (m). Imp: implant.

TEM was used for analyzing the ultrastructure of the cells located at the interface of gas voids. Most of the cells are multinucleated macrophages with several nuclei and resorbed Mg corrosion product in vacuoles and freely distributed in their cytoplasm (Figures 11(A)–11(C)). The multinucleated macrophages hold several long processes (Figure 11(C)). Besides multinucleated macrophages some macrophages with only one nucleus were found (Figures 11(E) and 11(F)). No resorbed Mg was detected in the cytoplasm of these cells. In addition some cells at the interface revealed a fibroblast like shape and exhibited an extended endoplasmatic reticulum (Figure 11(D)). No significant changes were determined in the ultrastructure of cells comparing the different Mg screws.

## 4. Discussion

In all magnesium implants a high amount of gas production with a consecutive bone defect (gas void) in the vicinity of the screw was detected after six and twelve weeks and one year. In all cases a proper osseous integration was not guaranteed but rather a loosening of the screw. On the other hand, all control samples, both PLLA and titanium, were already completely integrated after twelve weeks. The remodeling process of the bone tissue did not fill up the gas voids even after one year and connective tissue was still found inside the defect. The maximum observation period of twelve months was chosen, because the integration into the host bone of reconstructed ligaments is nearly finished after this time. However, in further studies it would be interesting to observe, if the connective tissue will be replaced by new-formed bone, beyond the twelve-month period.

The delayed osseous integration of all magnesium implants was confirmed by immunohistochemistry and molecular biology. The highest amount of multinuclear cells was found near the gas voids indicating a high activity of the tissue to control the gas release. This is confirmed by the higher amount of vessels in the granulation tissue and around the gas voids, seen in the ASMA and immunofluorescence. Nevertheless, the fact that the amount of vessels in the vicinity of gas voids decreased after 12 months indicates that the tissue reduced its effort to manage the gas liberation. Myofibroblasts were present in higher numbers after six weeks than after 12 weeks in the Mg groups compared to the control samples indicating replacement tissue rather than origin bone tissue. Furthermore transmission electron microscopy revealed the location of multinuclear cells in the vicinity of the gas voids with resorbed corrosion products. Only a few osteoclasts were detected at the trabecular bone representing the bone remodeling process. There was no significant difference between the different coating and producing methods. Huehnerschulte et al. [32] also found an increased number of osteoclasts in the vicinity of two magnesium alloys (ZEK100 and AX30) compared to the control samples concluding an adverse host reaction. Qi et al. [33] showed that PEO-coated magnesium implants did indeed have a decreased degradation in comparison to untreated implants but significantly more than the PLLA control sample. In contrast to other studies [7, 9], our results showed that the gas production process is still a crucial problem in the bone implantation site even after a calcium phosphate coating. The coated screws showed slightly bigger gas voids than the diecast and milled implants. In a recent study on PEO-coated ZK60 alloys an initially decreased degradation after 4 weeks was regained and passed after 12 weeks [31]. The coating was only able to reduce the degradation process in the first weeks compared to the bare implant. After that, the degradation was equal to that of the bare material or even surpassed it [15]. In our present study the PEO coating showed no difference after 6 and 12 weeks and one year. Coating only seems to delay the degradation process until the coating-layer is porous or absorbed [17]. Afterwards the degradation equals or even outstrips the bare metal situation. Perhaps a different alloy rather than coating could better control the degradation process and the following gas liberation for a longer time, since the interaction of a different alloy could reduce the gas liberation of the whole material and not only of its surface.

Regarding the production methods milled screws seem to induce less gas production than diecast screws. But, if we compare coated diecast screws with uncoated milled screws, a slightly lower gas formation can be observed in the former ones after one year.

There was evidence for foreign body reactions or infections in none of the implant regions verified by chloracetate esterase immunohistochemistry. Other studies confirm this result [32].

To elucidate the bone metabolism in the interface region in detail RT-PCR was conducted. Osteocalcin (OC) was significantly decreased in the coated implant group and in the diecast screws indicating less osteoanabolic activity in comparison to PLLA, titanium, and milled screws. Cathepsin K activity was also decreased in the coating group in relation to all other groups representing a less osteoclast activity. Even one year after implantation the expression of osteocalcin (OC), collagen-1 (Col1), and cathepsin K (CtsK) was lower in the magnesium group in comparison to PLLA screws. In summary, the coating seems to reduce the enzyme activity of the bone metabolism. Regarding the manufacture milling seems to have a better influence on bone metabolism than diecasting. Bondarenko et al. [34] also described a decreased osteocalcin activity in magnesium implants (LAE442) in comparison to titanium control samples after six months representing “a continuous degradation and instable bone-implant interface.” This confirms our result of decreased enzyme activity in the instable peri-implant region. Recent* in vitro* studies examined the cellular response on magnesium alloys and elucidated that a low concentration of about 2.5–10 mM Mg in the extracellular environment stimulate both osteoblasts and osteoclasts and hBMSC [35–37]. Furthermore, in a higher concentration magnesium chloride opposed cell proliferation and differentiation of osteoclasts [35]. Regarding our study we can assume that the increased magnesium chloride concentration resulting from the degradation processes in the extracellular matrix depressed cell proliferation and activity.

## 5. Conclusion

All W4 magnesium screws irrespective of the fabrication method or coating procedure showed a clinically relevant gas formation in the vicinity of the biomaterial. No screws, except the PLLA and titanium control samples, had any osseous integration in the surrounding tissue, resulting in a mechanically instable condition. Regarding the fabrication process, milling seems to have a lower gas liberation and a better influence on bone metabolism than diecasting. Coating by PEO with calcium phosphates could not sufficiently reduce the initial gas liberation but rather decreased the bone metabolism in the vicinity of the biomaterial. In summary, gas liberation in magnesium interference screws should be further reduced during the whole course of their degradation.

## Figures and Tables

**Table 1:** Allocation of the materials representing the different interference screws in relation to the different examination times. A total of 30 screws (two per animal) were used.

Interference screw	Fabrication procedure	6 weeks	3 months	12 months
PLLA		2	2	2

Titanium		2	2	—

W4, diecasted	*p* > 100 bar, *T* > 700°C;	2	—	—

W4, diecasted	*p* > 100 bar, *T* > 600°C; coating: CaO, CaHPO_4_, Mg(OH)_2_, nanohydroxyapatite (suspension), Ca(H_2_PO_4_), Na-silicate (Na_2_O·3SiO_2_)	—	2	—

W4, diecasted, coating no. 9	*p* > 100 bar, *T* > 600°C; coating: CaO, CaHPO_4_, Mg(OH)_2_, nanohydroxyapatite (suspension), Ca(H_2_PO_4_), Na-silicate (Na_2_O·3SiO_2_) zinkoxide	2	—	—

W4, diecasted, coating no. 8	*p* > 100 bar, *T* > 700°C; coating: CaO, CaHPO_4_, Mg(OH)_2_, nanohydroxyapatite (suspension), Ca(H_2_PO_4_), Na-silicate (Na_2_O·3SiO_2_)	2	—	—

W4, diecasted, coated 150 s	*p* > 100 bar, *T* > 600°C; coating: CaO, CaHPO_4_, Mg(OH)_2_, nanohydroxyapatite (suspension), Ca(H_2_PO_4_), Na-silicate (Na_2_O·3SiO_2_)	—	2	—

W4, diecasted, coated 300 s	*p* > 100 bar, *T* > 600°C; coating: CaO, CaHPO_4_, Mg(OH)_2_, nanohydroxyapatite (suspension), Ca(H_2_PO_4_), Na-silicate (Na_2_O·3SiO_2_)	—	2	2

W4, milled	Milled out of a two-step extruded cast	2	2	2

**Table 2:** List of primers for real-time RT-PCR.

Primer	Sequence 5′-3′	Length [bp^a^]	Accession number
*β*MG^b^			
fwd^c^	CCAGAAGATGGAAAGCCAAA	159	NM_0010009284
rev^d^	AGCGTGGGACAGAAGGTAGA		
Col1^e^			
fwd	CCAGTCACCTGCGTACAGAACG	245	AF129287
rev	GCCAGTGTCTCCTTTGGGTCC		
Bglap^f^			
fwd	CAGCGAGGTGGTGAAGAGAC	122	NM_001040009.1
rev	GCTCATCACAGTCAGGGTTG		
CtsK^g^			
fwd	GGGTCAATGTGGTTCCTGTT	133	XM004002465.1
rev	GCAGCCATCATTCTCAGACA		

^a^bp: base pair, ^b^ *β*MG: beta-2-microglobulin, ^c^fwd: forward, ^d^rev: reverse, ^e^Col1: collagen I*α*1, ^f^Bglap: gene of osteocalcin (OC), and ^g^CtsK: cathepsin K.

**Table 3:** Real-time RT-PCR.

	Control	Diecasting	Milling	Coating	1-year-Mg
Col1^b^	1.75 ± 0.51^a^	2.06 ± 0.45	3.76 ± 0.95	1.75 ± 0.47	−2.17 ± 1.35
		*P* = 0.571	*P* = 0.393	*P* = 0.429	*P* = 0.016^*^

OC^c^	−0.08 ± 0.53	−1.57 ± 0.47	−2.26 ± 1.97	−5.94 ± 1.25	−6.04 ± 1.25
		*P* = 0.036^*^	*P* = 0.25	*P* = 0.004^**^	*P* = 0.016^*^

CtsK^d^	1.6 ± 0.29	0.79 ± 0.66	0.54 ± 0.61	−0.42 ± 0.2	−0.7 ± 0.82
		*P* = 0.393	*P* = 0.143	*P* = 0.004^**^	*P* = 0.063

^a^Average ± standard error (SE), ^b^Col1: collagen-1, ^c^OC: osteocalcin, and ^d^CtsK: cathepsin K.

^*^ *P* ≤ 0.05.

^**^ *P* ≤ 0.01.
